# The Glasgow Prognostic Score Predicts Poor Survival in Cisplatin-Based Treated Patients with Metastatic Nasopharyngeal Carcinoma

**DOI:** 10.1371/journal.pone.0112581

**Published:** 2014-11-13

**Authors:** Cui Chen, Peng Sun, Qiang-sheng Dai, Hui-wen Weng, He-ping Li, Sheng Ye

**Affiliations:** 1 Department of Oncology, The First Affiliated Hospital, Sun Yat-Sen University, Guangzhou, China; 2 Department of Medical Oncology, Sun Yat-sen University Cancer Center, Guangzhou, China; 3 Collaborative Innovation Center for Cancer Medicine, State Key Laboratory of Oncology in South China, Guangzhou, China; University of Campinas, Brazil

## Abstract

**Background:**

Several inflammation-based prognostic scoring systems, including Glasgow Prognostic Score (GPS), neutrophil to lymphocyte ratio (NLR) and platelet to lymphocyte ratio (PLR) have been reported to predict survival in many malignancies, whereas their role in metastatic nasopharyngeal carcinoma (NPC) remains unclear. The aim of this study is to evaluate the clinical value of these prognostic scoring systems in a cohort of cisplatin-based treated patients with metastatic NPC.

**Methods:**

Two hundred and eleven patients with histologically proven metastatic NPC treated with first-line cisplatin-based chemotherapy were retrospectively evaluated. Demographics, disease-related characteristics and relevant laboratory data before treatment were recorded. GPS, NLR and PLR were calculated as described previously. Response to first-line therapy and survival data were also collected. Survival was analyzed in Cox regressions and stability of the models was examined by bootstrap resampling. The area under the receiver operating characteristics curve (AUC) was calculated to compare the discriminatory ability of each scoring system.

**Results:**

Among the above three inflammation-based prognostic scoring systems, GPS (*P*<0.001) and NLR (*P* = 0.019) were independently associated with overall survival, which showed to be stable in a bootstrap resampling study. The GPS consistently showed a higher AUC value at 6-month (0.805), 12-month (0.705), and 24-month (0.705) in comparison with NLR and PLR. Further analysis of the association of GPS with progression-free survival showed GPS was also associated independently with progression-free survival (*P*<0.001).

**Conclusions:**

Our study demonstrated that the GPS may be of prognostic value in metastatic NPC patients treated with cisplatin-based palliative chemotherapy and facilitate individualized treatment. However a prospective study to validate this prognostic model is still needed.

## Introduction

Nasopharyngeal carcinoma (NPC) is a distinct disease with unique ethnic and geographic characteristics, whose incidence varies from 0.5–3/100 000/year in North Africa to 20–30 in some areas of southern China. [1], [2] Although the cure rate has been significantly improved owing to advances in diagnostic imaging, radiotherapeutic techniques and chemotherapy regimens recently, distant metastases remain the main reason for failure of treatment. [3] In these cases, palliative systemic therapy remains the primary therapeutic option and cisplatin-based combination chemotherapy is considered the standard front-line regimen for decades, offering response rates in the range of 50–80% and a significant prolongation of overall survival (OS). [4] However, there are still wide individual differences in clinical response and outcomes. Some reports indicate that overall survival may exceed ten years for specific subgroups of patients. It is therefore of paramount interest to find an easily available model to help evaluate individual prognosis which will greatly improve the ability of clinical decision-making.

Currently, clinical characteristics are dominating indexes for judging prognosis of metastatic NPC patients, such as performance status and disease-free interval. [5] The prognostic value of circulating Epstein–Barr virus (EBV) DNA load has also been well established in various reports. [6], [7] Besides aforementioned prognostic factors representing tumor status and clinical characteristics, it is now recognized that the host inflammatory response, in particular the systemic inflammatory response, plays an important role in disease development and progression by inhibition of apoptosis, promotion of angiogenesis, and damage of DNA. [8], [9], [10] Several inflammation-based prognostic scoring systems have been devised and found to be strongly correlated with prognosis in patients with a variety of neoplasms. These include a combination of neutrophil and lymphocyte counts as the neutrophil to lymphocyte ratio (NLR) and a combination of platelet and lymphocyte counts as the platelet to lymphocyte ratio (PLR), both of which reflect full blood count derangements induced by the acute phase reaction, while the Glasgow Prognostic Score (GPS) incorporates raised circulating C-reactive protein (CRP) and hypoalbuminemia. [11], [12], [13], [14], [15] Recently some researches have also shown that markers of systemic inflammatory response represent reliable prognostic factors in patients with early nasopharyngeal carcinoma. [16] However, to the best of our knowledge, there is no data regarding the prognostic impact of systemic inflammation-based scoring systems in metastatic NPC. In the present study, we therefore evaluated the clinical value of several inflammation-based prognostic scoring systems including GPS, NLR and PLR in a cohort of cisplatin-based treated patients with metastatic NPC.

## Patients and Methods

### Patient selection

From October 2005 to October 2011, 211 patients with histologically proven metastatic NPC treated with first-line cisplatin-based chemotherapy were included in the study at Sun Yat-Sen University Cancer Center. Entry criteria consisted of: (1) radiologically measurable disease; (2) treated with at least two cycles of first-line cisplatin-based palliative chemotherapy; (3) Karnofsky Performance Scores (KPS) ≥60; (4) normal hepatic and renal function. Exclusion criteria included: (1) patients with other types of malignancy; (2) patients with brain metastases; (3) patients with clinical evidence of infection or other inflammatory conditions. This study was approved by the institutional review board and ethics committee of Sun Yat-Sen University Cancer Center. All patients provided written informed consent to participate in this study. Parental written consent was obtained for minors in current study.

### Treatment

All eligible patients received 1 of the following cisplatin-based chemotherapy regimens as the first-line treatment: (1) cisplatin (25 mg/m^2^ intravenously [IV] on Days 1–3 of a 21-day cycle) plus 5-fluorouracil (500 mg/m^2^ IV on Days 1–5 of a 21-day cycle), (2) paclitaxel (175 mg/m^2^ IV over 3 hours with standard premedication on Day 1 of a 21-day cycle) plus cisplatin (25 mg/m^2^ IV on Days 1–3 of a 21-day cycle), (3) paclitaxel (135 mg/m^2^ IV over 3 hours with standard premedication on Day 1 of a 21-day cycle) plus cisplatin (25 mg/m^2^ IV on Days 1–3 of a 21-day cycle) plus 5-fluorouracil (800 mg/m^2^, continuous IV infusion for 24 hours, on Days 1–5 of a 21-day cycle). Of the 211 eligible patients, 78 (37.0%) patients were given the PF regimen, 24 (11.4%) patients were given the TP regimen, and 109 (51.6%) patients received the TPF regimen.

### Relevant Evaluation

Basic demographics, baseline characteristics, detailed medical history as well as relevant laboratory data before treatment (C-reactive protein (CRP), Serum lactate dehydrogenase (LDH), albumin, neutrophil, lymphocyte, platelet (Plt) count and plasma EBV DNA level) were recorded. The GPS, NLR and PLR were constructed as described previously. In GPS, patients with both an elevated CRP level (>1.0 mg/dl) and hypoalbuminemia (<3.5 g/dl) were allocated a score of 2, patients with only one of these biochemical abnormalities were allocated a score of 1, and patients with neither of these abnormalities were allocated a score of 0. NLR was divided into two groups (<5 and ≥5) while PLR was categorized into three groups (<150, 150–300 and >300).

Progression-free survival (PFS) and overall survival (OS) were defined as the time from the first diagnosis of metastasis to the date of documented progression and to the date of death, respectively. Tumor response was evaluated according to the Response Evaluation Criteria in Solid Tumors (RECISTs) 1.0.

### Follow up

Patients were regularly followed up after chemotherapy until death or their last follow-up appointment. Physical examination and imaging studies of the relevant region(s) were performed every 3 months after the completion of the chemotherapy or when clinical indications dictated for follow-up. The start date of follow-up period was the date of initial metastatic NPC diagnosis. The time of last follow-up was 31st December 2013 or death.

### Statistical analysis

All statistical analysis was performed using SPSS version 13.0 software or WinStat software. PFS and OS were obtained by using the Kaplan–Meier method and differences between the groups were compared by the log-rank test. A univariate analysis was performed for the potential prognostic factors. Age, karnofsky performance score before treatment, number of involved sites, disease-free interval, serum LDH, pre-treatment EBV DNA entered the calculations in a continuous way. NLR and PLR were also tested at first as continuous variables in order to avoid the bias induced by binarization of continuous data. And we tested the GPS and the other variables entering the analysis as categorical variables. Multivariable analysis including variables that proved to be significant in the univariate analysis was performed subsequently using the Cox model to analyse factors related to prognosis (P<0.05 was used as the cut-off value of statistical significance). The stability of the COX model was tested by bootstrap resampling. New data sets of equal size were created by random sampling of the original data with replacement. In each new bootstrap data set, a patient may be represented once, multiple times or not at all. Cox regressions with the same conditions as in the original data set were then calculated for the new data sets in order to obtain the bootstrap parameter estimates. Descriptive statistics for the patient groups are reported as mean, median, and range. Categorical variables were presented numbers and percentages. Non-parametric test was applied for comparison of data among groups. A receiver operating characteristics (ROC) curve was also generated and the area under the curve (AUC) was calculated to evaluate the discriminatory ability of each scoring systems. A two-tailed P value less than 0.05 was considered to be statistically significant.

## Results

### Patient characteristics and Outcomes

A total of 211 patients with metastatic NPC were included in the present study. All of the patients were from epidemic areas in China, with a male predominance (85.8%). The mean age of diagnosis of metastatic NPC was 46 (range 14–72) years. About half of the patients had more than one metastatic site with lung being the most common site (45.9%). The pretreatment plasma EBV DNA ranged from 0 to 9.73×10^7^ copies/mL, with a median of 4.93×10^4^copies/mL. One hundred and fifty (71.1%) patients showed an elevated pretreatment EBV DNA level (>1×10^3^copies/mL). One hundred and twenty-five (59.2%) patients were allocated to GPS 0, 66 (31.3%) patients were allocated to GPS 1, and 20 (9.5%) patients were allocated to GPS 2, respectively. The median NLR level was 3.12 (range 0.81∼11.03). Thirty patients (14.2%) had an NLR≥5 and the rest had an NLR<5. The PLR ranged from 31.3 to 422.5, with a median of 71.2. A PLR greater than 300 was seen in 5 patients (2.4%), 168 patients (79.6%) had PLR<150, and the rest had a PLR in between. Other patient characteristics are summarized in Table 1.

**Table 1:** Demographic and Baseline Characteristics of Patients.

Patient characteristics	Number (%)
Total evaluated	211 (100)
Age, years (median/range)	46/14–72
Gender (male/female)	181/30 (85.8/14.2)
KPS (median/range)	90/60–100
Number of involved sites (median/range)	2/1–6
Synchronous metastasis (yes/no)	53/158 (25.1/74.9)
Liver metastasis (yes/no)	73/138 (34.6/65.4)
Lung metastasis (yes/no)	97/114 (45.9/54.1)
Bone metastasis (yes/no)	88/123 (41.7/58.3)
Disease-free interval, months (median/range)	6/0–65
Chemotherapy regimen (PF/TP/TPF)	78/24/109 (37.0/11.4/51.6)
Serum LDH, U/L (median/range)	247/81–632
Pre-treatment EBV DNA, copies/mL (median/range)	4.93×10^4^/0–9.73×10^7^
GPS (0/1/2)	125/66/20 (59.2/31.3/9.5)
NLR (median/range)	3.12/0.81–11.03
PLR (median/range)	71.2/31.3–422.5

At the time of analysis, 124 (58.8%) patients had died, and the median PFS and OS were 7.9 and 21.6 months, respectively. The overall clinical response rate was 70.1% for all 211 patients.

### Prognostic factor analysis for overall survival

**Figure 1:**
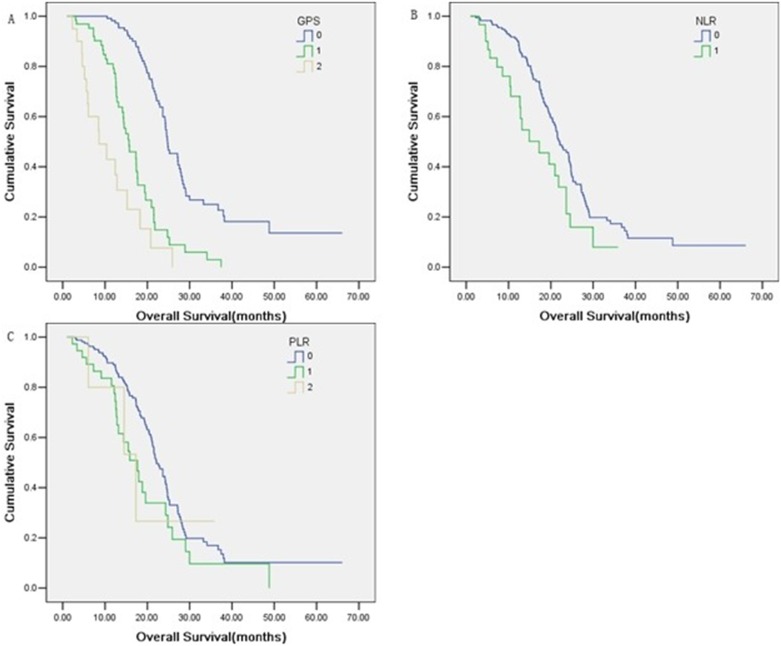
Comparison of overall survival according to scoring systems, GPS (A), NLR (B) and PLR (C).

Various potential prognostic factors including age, gender, karnofsky performance score before treatment, metastasis sites (liver and lung), number of involved sites, synchronous metastasis, disease-free interval, chemotherapy regimen, serum LDH, pre-treatment EBV DNA, GPS status, NLR and PLR were analyzed. Univariate analysis revealed that a larger number of involved sites (*P* = 0.020), higher baseline serum LDH level (*P* = 0.014), higher pretreatment EBV DNA level (*P* = 0.024), higher score of GPS (*P*<0.001) and higher value of NLR (*P* = 0.025) were considered adverse factors for overall survival (Table 2, Fig. 1). Age, gender, PLR and the other variables in the analysis had no prognostic relevance. In multivariate analysis, pre-treatment EBV DNA (*P* = 0.037), GPS (*P*<0.001) and NLR (*P* = 0.019) were independent prognostic factors (Table 2). The stability of this model was confirmed in a bootstrap resampling procedure. Among 1000 new models, pre-treatment EBV DNA was present in 69%, GPS appeared in 89% and NLR in 71%.

**Table 2:** Univariate and Multivariate Analysis of Prognostic Factors of Overall Survival.

	Univariate analysis		Multivariate analysis	
Variable	*P*	HR (95% CI)	*P*	HR (95% CI)
Age	0.444	1.006 (0.990–1.023)		
Gender (male/female)	0.631	1.147 (0.655–2.008)		
KPS	0.934	1.020 (0.637–1.633)		
Liver metastasis (yes/no)	0.989	1.003 (0.694–1.449)		
Lung metastasis (yes/no)	0.848	1.035 (0.726–1.476)		
Number of involved sites	0.020	1.282 (1.040–1.580)	0.560	1.064 (0.864–1.310)
Synchronous metastasis(yes/no)	0.696	0.920 (0.604–1.400)		
Disease-free interval	0.278	1.218 (0.853–1.739)		
Chemotherapy regimen(PF/TP/TPF)	0.358	0.767 (0.435–1.351)		
Serum LDH	0.014	1.210 (1.040–1.409)	0.911	1.011 (0.835–1.225)
Pre-treatment EBVDNA	0.024	1.234 (1.028–1.481)	0.037	1.239 (1.013–1.515)
GPS (0/1/2)	<0.001	3.078 (2.393–3.959)	<0.001	2.520 (1.977–3.212)
NLR	0.025	1.732 (1.071–2.800)	0.019	1.800 (1.103–2.940)
PLR	0.125	1.311 (0.928–1.853)		

**Figure 2:**
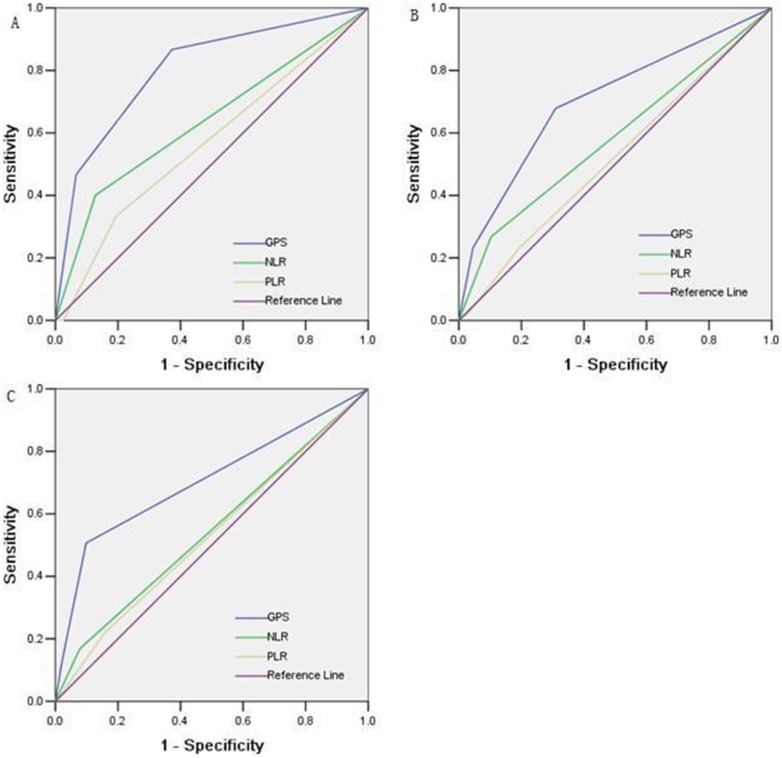
Comparisons of the area under the receiver operating curve for survival status between scoring systems at 6 month (A), 12 month (B) and 24 month (C).

Moreover, the two inflammation-based prognostic scoring systems constructed by categorizing the continuous variables of NLR and PLR as described before were compared with the GPS. Receiver operating characteristic curves were constructed for survival status at 6-month, 12-month, and 24-month of follow-up, and the area under the ROC curve (AUC) was compared (Fig. 2) to assess the discrimination ability of each scoring system. The GPS consistently show a higher AUC value at 6-month (0.805), 12-month (0.705), and 24-month (0.705) in comparison with other inflammation-based prognostic scores.

### Association of GPS with clinicopathologic characteristics

Baseline patient and disease-related characteristics for each GPS group and comparisons between groups are depicted in Table 3. Although the difference was not statistically significant, a trend towards an association of GPS with BMI was observed. Of note, an elevated GPS was significantly associated with higher serum LDH and higher pretreatment EBV DNA.

**Table 3:** Association of GPS with characteristics of patients.

characteristics	GPS = 0	GPS = 1	GPS = 2	*P*
Age (≤45/>45)	65/60	30/36	12/8	0.472
Gender (male/female)	103/22	60/6	18/2	0.236
KPS (≤80/>80)	24/101	12/54	4/16	0.978
BMI (≤18.5/>18.5)	21/104	20/46	6/14	0.070
Number of involved sites (1/≥2)	61/64	36/30	8/12	0.493
Synchronous metastasis (yes/no)	26/99	22/44	5/15	0.165
Liver metastasis (yes/no)	43/82	21/45	9/11	0.553
Lung metastasis (yes/no)	53/72	35/31	9/11	0.373
Bone metastasis (yes/no)	50/75	28/38	10/10	0.694
Serum LDH, U/L (<245/≥245)	83/42	27/39	8/12	0.001
Pre-treatment EBV DNA, copies/mL (<median/≥median)	99/26	5/61	2/18	0.0001

### Association of GPS with progression-free survival

**Figure 3:**
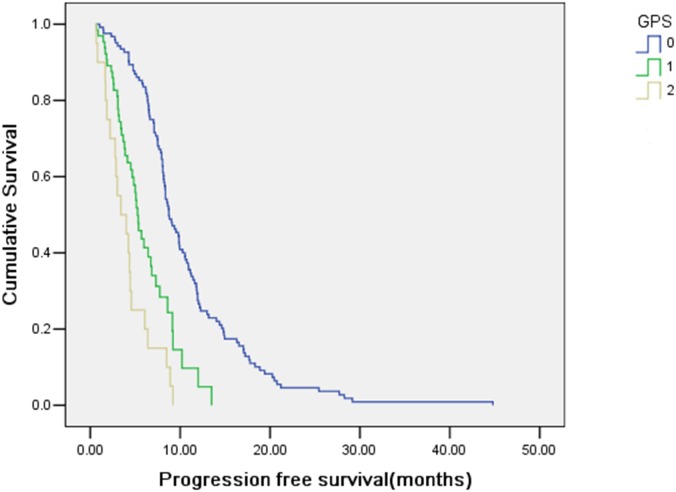
Kaplan–Meier estimates for progression-free survival according to GPS.

GPS was further associated with PFS. Kaplan–Meier curves for PFS for the total cohort according to GPS was shown in Fig. 3. Median PFS (95% CI) was 8.73 (7.64–9.82), 5.27 (4.51–6.02) and 3.40 (1.21–5.59) months for patients with GPS 0, 1 and 2, respectively. As shown in Table 4, multivariate analysis including the aforementioned parameters and GPS revealed that GPS was also the independent predictor for PFS (*P*<0.001). The stability of this model was also confirmed in a bootstrap resampling procedure. In the bootstrap resampling, GPS entered in 100% and pre-treatment EBV DNA appeared in 25%.

**Table 4:** Univariate and Multivariate Analysis of Prognostic Factors of Progression-free Survival.

	Univariate analysis		Multivariate analysis	
Variable	*P*	HR (95% CI)	*P*	HR (95% CI)
Age	0.613	0.996 (0.981–1.011)		
Gender (male/female)	0.489	1.162 (0.760–1.776)		
KPS	0.372	0.998 (0.994–1.002)		
Liver metastasis (yes/no)	0.477	0.893 (0.655–1.219)		
Lung metastasis (yes/no)	0.127	1.261 (0.936–1.698)		
Number of involved sites	0.043	1.201 (1.0066–1.435)	0.493	1.063 (0.893–1.266)
Synchronous metastasis (yes/no)	0.933	1.015 (0.719–1.434)		
Disease-free interval	0.238	1.198 (0.887–1.617)		
Chemotherapy regimen (PF/TP/TPF)	0.609	0.884 (0.552–1.417)		
Serum LDH	0.340	1.072 (0.93–1.235)		
Pre-treatment EBVDNA	<0.001	1.426 (1.170–1.739)	0.133	1.206 (0.945–1.539)
GPS (0/1/2)	<0.001	2.417 (1.916–3.050)	<0.001	2.248 (1.753–2.833)
NLR	0.054	1.400 (0.995–1.971)		
PLR	0.611	1.061 (0.844–1.334)		

## Discussion

Markers of systemic inflammatory response represent reliable prognostic factors in patients with advanced cancer. [8], [11], [12], [13], [14], [16] To the best of our knowledge, this study has firstly demonstrated that the GPS, an inflammation-based prognostic score, is an independent marker of poor prognosis in patients with metastatic NPC and is superior to the NLR in terms of prognostic ability. Furthermore, our data demonstrated a significant, independent association between GPS and PFS.

Accumulating evidence indicates the prognostic importance of GPS in various solid cancers, such as colorectal cancer, [17], [18] esophageal cancer, [19] lung cancer, [13] pancreatic cancer, [12] and gastric cancer. [14] A similar result was achieved in our study. The biological basis for the correlation between the GPS and survival are not completely understood. Below are some supposed mechanisms. First, cachexia, which often manifests as nutritional depletion (weight loss, elevated resting energy expenditure and loss of lean tissue) and functional decline, is common in patients with advanced cancer and has been recognized to be associated with poorer outcome. [20], [21], [22] CRP has been reported to be associated with the nutrition status and development of cachexia while albumin represents a negative acute phase protein and also represents a marker of nutritional status. [8] As we know, lower serum albumin correlates to nutritional depletion closely. Our study also shows a trend towards an association of GPS with BMI. Based on these reports, GPS, incorporating CRP and serum albumin levels, may reflect both presence of the nutritional depletion and functional decline, resulting in poor survival outcome. Second, a strong association was found between EBV infection and NPC in previous studies. [23] Plasma EBV DNA has been identified to be prognostic in metastatic NPC patients. [6], [7] EBV infection stimulated the release of pro-inflammatory cytokine including IL-1, IL-6, and TNF-α from the tumor microenvironment, which results in the induction of CRP synthesis from the liver and the reduction of albumin by hepatocytes. [24], [25] In other words, GPS level may be a marker of inflammation from EBV infection and may indicate the magnitude of inflammation and the prognosis of patients as EBV DNA load. Previous studies have also indicated that inflammation in the tumor microenvironment play an important role in promoting tumor growth, invasion, and metastasis. [9], [10] Our data shows that an elevated GPS is significantly associated with higher EBV-DNA level, which will, to certain extent, add further support to the proposal. In addition to these explanations, because our data find an elevated GPS is also significantly associated with elevated LDH, which has been reported to be an indicator of high tumor burden, an elevated GPS score may indirectly reflect a high tumor burden. [26] In general, these explanations suggest that it is reasonable that GPS is a significant and independent predictor of survival outcome.

Recently a study by Wei-xiong Xia et al also showed that elevated CRP and CRP kinetics correlated with poor prognosis in patients with metastatic NPC. This study had similar aims and results compared with our study. However there are still some differences between the two studies. Firstly, the GPS incorporates CRP and hypoalbuminemia and may be more suitable to reflect systemic inflammatory response than CRP alone. Secondly, the eligibility criteria are different. All patients enrolled in current study received first-line cisplatin-based regimens. Thus, it is helpful to exclude the potential confounding effect of different regimens.

The GPS test is simple and based on standardized, wildly available protein assays. Therefore assessment of the GPS can be routinely in most clinical centers. Based on the present results, the significant value of GPS test is that it can identify patients at high risk of disease progression and death as a clinically convenient and useful biomarker. Thus it not only provides guidance of follow-up care at clinic but also has the potential to be a stratification factor or a selection criterion in randomized clinical trials for metastatic NPC. Moreover, in our study, most of the patients evaluated as disease progression at the end of second cycle of chemotherapy were allocated a score of 2. Patients in the good GPS group (GPS 0) had a more prolonged progression-free survival. As a consequence we believe that the presence of a systemic inflammatory response should be evaluated in the pretreatment period and might become the promising new targets of anti-tumor therapy. Nowadays there was an amount of ongoing research into the effect of non-steroidal anti-inflammatory drugs on anti-tumor treatment, including colon cancer, [27] lung cancer, [28] esophagus cancer [29] and so on. Accordingly, it is also interesting and significant to study the modification of the systemic inflammatory response in patients with metastatic nasopharyngeal carcinoma. And the GPS which is inexpensive, reliable, and widely available may have a certain guiding significance for selecting patients who might be candidates for modulation of systemic inflammatory response and provide a well defined therapeutic target for future clinical trials. Further evaluation is required to confirm this hypothesis.

In addition, the NLR and PLR have been reported to be important prognostic models in patients with a variety of solid cancers, such as colorectal cancer, esophageal cancer, gastric cancer, pancreatic cancer, and lung cancer. Several studies have also shown that an elevated NLR is associated with poor prognosis in patients with NPC. [12], [14], [30], [31] In accord with the study of Jian-rong He et al. who tested the prognostic value of NLR in 1410 patients with various stages of NPC [32] and the study of Xin An et al. who tested the prognostic value of NLR in 363 patients with non-disseminated NPC, [16] we also found a significant association between NLR and OS. However, the COX model and the AUC analysis have shown that the GPS was superior to NLR in terms of discriminating ability and prognostic accuracy. For PLR, it was not independently associated with overall survival. In general, this study is the first to show the superior prognostic ability of the GPS over the NLR and PLR in patients with metastatic NPC.

In conclusion, our study demonstrated that the GPS may be useful to predict the prognosis of metastatic NPC patients treated with cisplatin-based palliative chemotherapy and facilitate individualized treatment. A prospective study to validate this prognostic model is needed. The mechanisms underlying the relationship between high GPS and poor prognosis in NPC still need further study.
